# Effects of short-term moderate versus high intraocular pressure elevation on flicker-induced changes in full-field electroretinogram

**DOI:** 10.1007/s10633-026-10129-3

**Published:** 2026-07-04

**Authors:** Milan Rai, Xiayin Yang, Kai Yip Choi, Shaoying Tan, Henry Ho-lung Chan

**Affiliations:** 1 School of Optometry, The Hong Kong Polytechnic University, Kowloon, Hong Kong SAR China; 2 Laboratory of Experimental Optometry (Neuroscience), School of Optometry, The Hong Kong Polytechnic University, 11 Yuk Choi Road, Hung Hom, Kowloon, Hong Kong SAR China; 3 Centre for Eye and Vision Research (CEVR), 17W Hong Kong Science Park, Hong Kong, Hong Kong SAR China; 4 Colour Imaging and Metaverse Research Centre, The Hong Kong Polytechnic University, Kowloon, Hong Kong SAR China

**Keywords:** Flickering light stimulation, Middle retinal layer, Vascular loop, Ocular perfusion pressure, Electrical activity

## Abstract

**Purpose:**

Transient flickering light stimulation (FLS) enhances the electrical activity in the middle retinal layer (MRL) of wild-type mice. This study investigates how short-term moderate and high intraocular pressure (IOP) elevation influences flicker-induced enhancement in the retinal activity using full-field electroretinograms (ffERG).

**Methods:**

Baseline blood pressure (BP), IOP, mean ocular perfusion pressure (OPP), and ffERG were measured before and after FLS in eighteen C57BL/6 J mice. The mice were subsequently divided into two groups: ocular hypertension (OHT, n = 9) and control (n = 9). In the OHT group, IOP was firstly transiently elevated to ~ 35 mm Hg for 5 min (termed as Loop on Phase-1 (LOP-1)) using an adjustable vascular loop in one randomly chosen eye, while the control group had the loop placed without IOP elevation. IOP was further elevated to ~ 65 mm Hg in the same eye for another 5 min (termed as Loop on Phase-2 (LOP-2)) in the OHT group, while the control group had the loop placed in the same eye without IOP increase. The BP, IOP, mean OPP and ffERG measurements were repeated before and after FLS in each condition.

**Results:**

While BP showed no significant differences, mean OPP was significantly reduced at LOP-1 and LOP-2 in the OHT group compared to both baseline (*p* < 0.001) and to the control group (*p* < 0.001). The b-wave amplitudes recorded after FLS were significantly higher than those before FLS at baseline and LOP-1 conditions in both control (*p* < 0.01) and OHT groups (*p* < 0.001). In the LOP-2 condition, the OHT group showed no significant difference (*p* > 0.05) between pre- and post-FLS b-wave amplitudes, while the control group exhibited a significant increase (*p* < 0.001). The percentage change in b-wave amplitude was significantly reduced in the OHT group at LOP-2 condition (Pre-Loop vs LOP-2: *p* < 0.01; LOP-1 versus LOP-2: *p* < 0.001), while the control group maintained a consistent percentage increase in b-wave amplitudes. No such significant changes were found in other parameters of ffERG response after FLS.

**Conclusions:**

Short-term high IOP elevation (~ 65 mm Hg), but not moderate (~ 35 mm Hg), disrupted flicker-induced enhancement of MRL electrical activity. This implies that the retina can adapt to a short period of moderate IOP, sustaining a normal increase in the retinal electrical activity in response to FLS. However, even a short period of high IOP would cause certain physiological damage to the retina.

**Supplementary Information:**

The online version contains supplementary material available at https://doi.org/10.1007/s10633-026-10129-3.

## Introduction

Elevated intraocular pressure (IOP) is one of the most well-documented risk factors for glaucoma, a leading cause of irreversible blindness worldwide [1, 2]. Increased IOP causes direct mechanical damage to retinal ganglion cell axons [3] and may also shear astrocyte attachments from the optic nerve head, resulting in the loss of metabolic support [4]. Moreover, the compression of blood vessels, caused by elevated IOP, may lead to insufficient blood supply, resulting in oxygen and nutrients deprivation [5]. This deficiency can induce ischemic changes in the retina and optic nerve head, facilitating the progression of glaucomatous damage [5, 6]. In addition, the compromised retinal blood flow can adversely alter retinal perfusion pressure, potentially affecting retinal neurovascular coupling (the mechanism by which increased retinal neural activity from light stimuli leads to dilation of retinal vessels and enhanced blood flow) and impairing normal retinal physiology [7]. Several studies have widely reported the diminishing effects of increased IOP on the structure and function of the retina [8–11]. Currently, the assessments of retinal layer thickness (specifically the retinal nerve fibre layer thickness), optic nerve head appearance, visual fields, and electrophysiological responses are conducted to detect retinal structural and functional changes caused by elevated IOP. However, the changes, such as retinal nerve fibre layer thinning and visual field defects, are often only detectable and measurable once the cumulative damage reaches a certain threshold [3, 12]. Recent studies have reported that flicker-evoked increases in retinal blood flow (RBF) and blood vessel diameter are reduced in glaucoma patients compared to healthy subjects [13–16]. Specifically, Riva et al. reported a significant reduction in flicker-induced retinal blood flow responses at the optic disc rim in early open-angle glaucoma and ocular hypertensive patients [13]. Garhofer et al. found a significant reduction in flicker-induced vasodilation of retinal veins in patients with early glaucoma [14]. Gugleta et al. reported an impaired flicker-evoked retinal vessel response (vasodilation) in the primary open angle-glaucoma patients [15, 16]. These findings suggest that the hemodynamic component of the retinal neurovascular coupling could be impaired by elevated IOP.

Although previous studies have assessed the flicker-induced changes in retinal vascular calibres, such as RBF and vasodilation in glaucoma and ocular hypertensive patients [13–16], the effect of elevated IOP on flicker-induced changes in retinal electrophysiological responses (representing neuronal component of neurovascular coupling) is still unknown. Recently, we have shown that retinal stimulation with flickering light can transiently enhance electro-retinal responses in addition to an increase in RBF in healthy mice [17]. Our findings suggest that both hemodynamic and neuronal components are transiently enhanced by retinal stimulation with flickering light stimulation (FLS).

Although we have assessed and quantified the changes in ffERG responses due to transient FLS in wild-type mice, the influence of short-term IOP elevation on these changes remains unclear. Therefore, this study aims to investigate the effects of short-term moderate (~ 35 mm Hg) and high (~ 65 mm Hg) IOP elevations on flicker-induced alterations in ffERG responses in a mouse model.

In the present study, our primary hypothesis is that short-term IOP elevation would attenuate the flicker-induced increases in ERG responses as elevated IOP has been found to impair the retinal vascular autoregulation [18]. To test this hypothesis, we measured the retinal neuronal responses before and after FLS using our reported full-field electroretinogram (ffERG) protocols [17] at two different levels of IOP (~ 35 mm Hg, and ~ 65 mm Hg) and compared the effects of these varying IOP levels on flicker-induced ffERG changes in mice.

## Methods

### Animals

All experimental procedures were carried out in adherence to the ARVO Statement for the Use of Animals in Ophthalmic and Vision Research. The study protocol was approved by the Animal Ethics Sub-committee of The Hong Kong Polytechnic University (ASESC No.:18-19/58-SO-R-OTHERS). Eighteen C57BL/6 J mice (age: 8 weeks, 10 males and 8 females), were housed in the centralised animal facilities in a temperature-controlled room (21–22 °C) under normal lighting condition (approximately 200 lx) with a 12/12-h light/dark cycle. Mice were provided with unrestricted access to food (Pico Lab diet 20 (5053); PMI Nutrition International, Richmond, IN, USA) and water.

### Experimental design

**Fig. 1:**
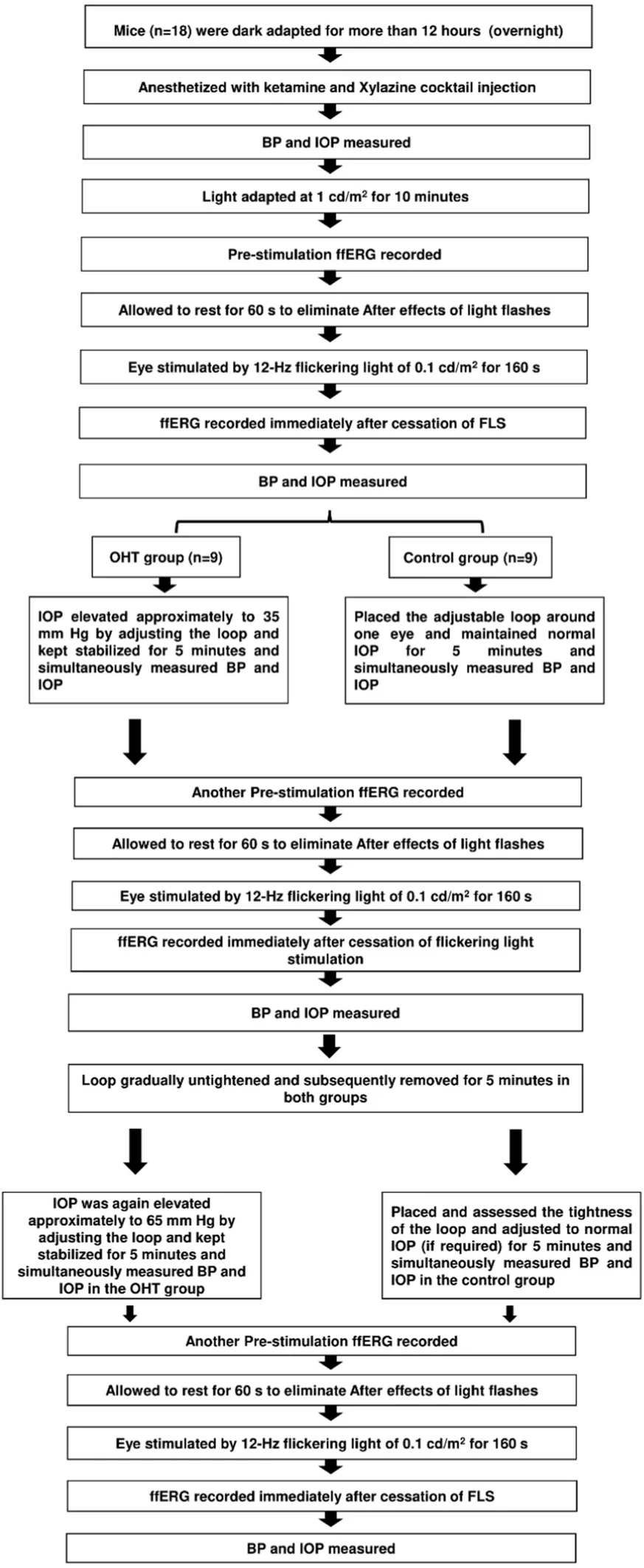
Flow diagram showing the experimental protocol of the study

Following overnight dark adaptation for more than 12 h, mice were anesthetized via intraperitoneal injection of a cocktail containing 90 mg/kg of 10% ketamine (Alfasan International B.V., Woerden, Holland) and 12 mg/kg of 2% xylazine (Alfasan International B.V.). All eighteen mice underwent baseline blood pressure (BP) and IOP measurements. Then, ffERGs were recorded in all mice before and after FLS according to our establish bed experimental protocols [17]. BP and IOP measurements were subsequently repeated. Following that, mice were randomly assigned to either the OHT group (n = 9) or the control group (n = 9). In the OHT group, IOP was elevated to approximately 35 mm Hg by adjusting a ligature (adjustable vascular loop) placed around one randomly chosen eye. This elevated IOP was maintained for 5 min, during which BP and IOP were measured. After the stabilization period, ffERG measurements were repeated (under elevated IOP condition) before and after FLS, following the same experimental protocol as described above. BP and IOP measurements were then repeated. Thereafter, the loop was gradually untightened and subsequently removed for 5 min to minimise the effects of elevated IOP caused by loop tightening. Following this resting period of about 5 min, IOP was further elevated to approximately 65 mm Hg by gradually tightening the ligature. This elevated IOP was also sustained for 5 min, during which BP and IOP measurements were repeated. After stabilization, ffERG recordings were again repeated (under elevated IOP condition) before and after FLS, as previously described, followed by BP and IOP measurements. In contrast, the control group underwent the same experimental protocols but without any elevation of IOP, thereby maintaining normal IOP throughout the entire procedure. To minimise the potential confounding effects of circadian rhythms, all experiments for both experimental groups were conducted between 12:00 PM and 5:00 PM. A flowchart depicting the experimental steps is presented in Fig. 1.

### IOP manipulation

**Fig. 2:**
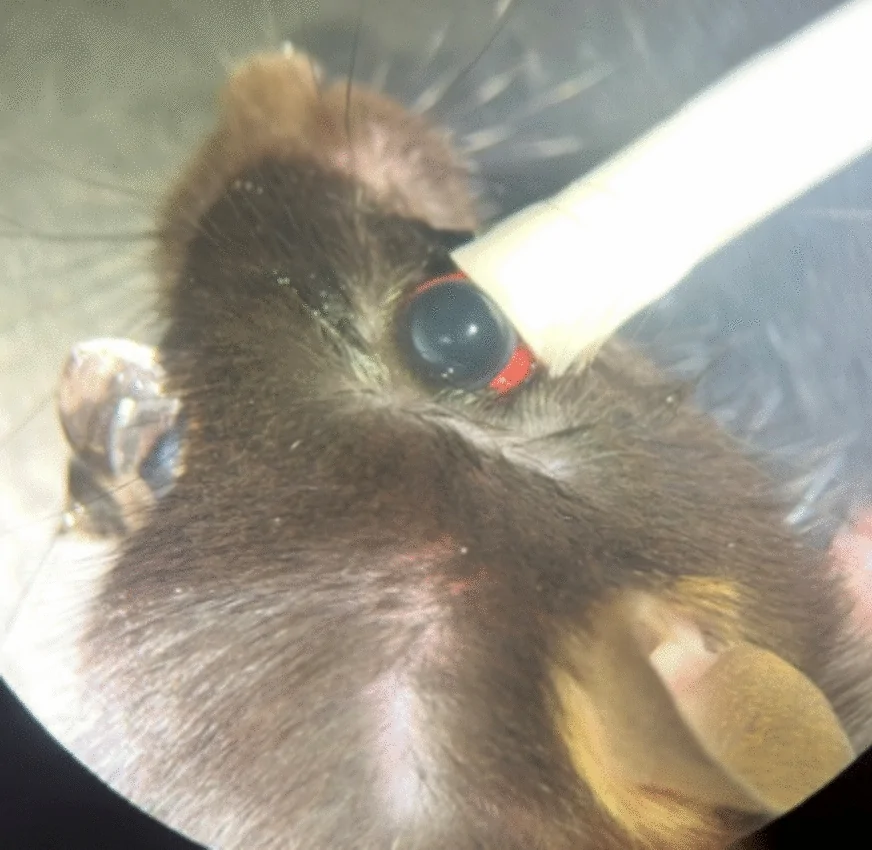
A photograph depicting an adjustable vascular loop placed around the mouse eye

The procedures for inducing IOP elevation have been described in the previous study [19]. IOP was manually elevated by using an adjustable loop placed around one randomly chosen eye. The adjustable loop consisted of an 8-cm long small vascular loop (RS PRO Mains 5 Core Power Cable, Red PVC) measuring 1.5 mm in diameter, which was inserted within a plastic tubing (weight: 1.4 g) with a loop of 2 mm internal diameter and a length of 4 cm. The loop was placed anterior to the equator of the eye following the topical administration of local anaesthesia (1% tetracaine; Alcon Laboratories, Ft. Worth, TX, USA). The desired IOP elevation was induced by gradually tightening the vascular loop (~ 5 mm Hg increase in ~ 10 s). In the OHT group, the loop was gradually adjusted to elevate IOP at approximately 35 mm Hg during the first phase and at around 65 mm Hg in the second phase. In the control group, the loop was placed without tightening to keep IOP within the normal range throughout both phases. The condition prior to the application of the loop is referred to as Pre-Loop (baseline). The initial phase of loop adjustment is termed as Loop on Phase-1 (LOP-1), while the second phase of adjustment is termed as Loop on Phase-2 (LOP-2). In the OHT group, these phases correspond to the adjustments made to maintain IOP at 35 mm Hg (LOP-1) and 65 mm Hg (LOP-2), respectively. The IOP was measured using the Tonolab tonometer TV02 (ICare, Vantaa, Finland). Additional local anaesthesia (1% tetracaine; Alcon Laboratories, Ft. Worth, TX, USA) was applied to the eye every 15 min after loop placement until the end of experiment. An Image captured during the placement of adjustable vascular loop around an eye of a mouse is shown in Fig. 2.

### Blood pressure measurement

Blood pressure was measured using a non-invasive automated tail-cuff system (Vistech BP-2000 Blood Pressure Analysis System; Vistech Systems; Apex, NC) [20, 21]. Mean OPP was calculated as the difference between mean BP and IOP, which is different from the relationship used in humans (OPP = (2/3) MAP-IOP) [22]. As the ‘2/3’ MAP adjustment factor is only applicable to rodents in an upright position, it was not incorporated in our study, given that mouse BP was measured in a prone position (anesthetized) [22].

### Full-field electroretinogram

The procedures and protocols used for recording ffERG measurements were described in detail elsewhere [17]. Briefly, the ffERG recordings were performed using a Ganzfeld ERG system (Q450; RETI Animal, Roland Consult, Brandenburg an der Havel, Germany). After applying a drop of Provain-POS 0.5% (URSAPHARM, Saarbrücken, Germany) and Mydriacyl 1% (Alcon- Couvreur, Puurs, Belgium) to the eye for anesthesia and pupil dilation respectively, the animal was positioned on a heating pad to maintain its body temperature around 37 °C. Lacryvisc gel (Alcon, Rueil-Malmaison, France) was uniformly spread over the cornea to prevent dehydration and opacification of the lens caused by anesthesia-induced optical changes. A 2-mm-diameter gold ring electrode (Roland Consult) was placed on the cornea of each eye for ffERG recording. Reference and ground electrodes were needle electrodes (Item No. U51-426; GVB-geliMED, Bad Segeberg, Germany) inserted into the lateral canthus of each eye and the upper base of the tail, respectively. Impedance was below 5 KΩ for all recordings. Following a 10-min light adaptation at a background luminance of 1 cd/m^2^, ffERG responses (25 responses, interstimulus interval of 1 s) were subsequently measured by presenting a brief white LED flash (duration: 4 ms) at an intensity of 3.0 cd·s/m^2^. Subsequent to flash stimulation, the animals were allowed to rest for 60 s to minimise the aftereffects of the flashes. A 12 Hz square-wave flickering light (with a white LED source at an intensity of 0.1 cd·s/m^2^) was used to stimulate the retina for 160 s. The ffERG measurement was immediately repeated following the cessation of the FLS. The amplitudes and implicit times of the a-wave and the b-wave were extracted for analysis.

### Data analysis

Data are presented as mean and standard error of the mean (SEM). The mixed-model ANOVA was conducted to assess differences in BP, IOP, OPP, and ffERG responses between the two groups, and within groups, with post-hoc Bonferroni correction for multiple comparisons. The relative changes in ffERG responses at each IOP level are presented as percentage changes from their respective baseline values [(Post FLS–Pre FLS)/Pre FLS]. The mixed-model ANOVA with Bonferroni post-hoc tests were used to compare these percentage changes in flicker-induced ffERG within and between groups. All statistical analyses were performed using SPSS 30.0 (IBM Corp. Armonk, NY, USA). A *p*-value of less than 0.05 was considered statistically significant.

## Results

The mixed-model ANOVA showed significant effects for both within-group and between groups comparisons, as well as interaction effects for IOP and OPP, but not for BP as shown in Table 1. At baseline (both before and after FLS), there were no significant differences in IOP and OPP between the two groups. However, following loop adjustments, the OHT group showed significantly elevated IOP and reduced OPP compared to the control group in both LOP-1 and LOP-2 conditions (both pre- and post-FLS). There were no significant differences in BP, which remained stable over time both between and within the groups. Table 1 presents the mean (SEM) values of IOP, BP, and OPP for the two experimental groups at baseline and Loop on phases obtained before and after FLS. The individual mean values of BP, IOP, and OPP data obtained from both groups are presented in the Supplementary Material (ESM_1).

**Table 1:** Mean (SEM) values of IOP (mm Hg), BP (mm Hg), and OPP (mm Hg) measured before and after FLS in the OHT and control groups at Pre-Loop (Baseline), LOP-1, and LOP-2 conditions

Parameters	Group	Pre-loop before FLS	Pre-loop after FLS	LOP-1 before FLS	LOP-1 after FLS	LOP-2 before FLS	LOP-2 after FLS	Within-group main effect	Between-groups main effect	Interaction effect	Between groups comparisons					
											Pre-loop(baseline) beforeFLS	Pre-loop(baseline) after FLS	LOP-1 before FLS	LOP-1 after FLS	LOP-2 before FLS	LOP-2 after FLS
IOP(mm Hg)	OHT	11.67 (0.82)	11.67 (0.91)	36.11 (1.06)	34.44 (1.32)	65.00 (0.75)	64.33 (1.75)	** < 0.001**	** < 0.001**	** < 0.001**	> 0.05	> 0.05	** < 0.001**	** < 0.001**	** < 0.001**	** < 0.001**
	Control	12.78 (0.83)	13.56 (1.03)	15.22 (0.62)	15.22 (0.91)	14.22 (0.46)	14.44 (0.65)									
BP (mm Hg)	OHT	73.61 (1.66)	72.09 (1.41)	73.05 (1.03)	71.77 (1.18)	73.57 (1.36)	74.16 (1.17)	> 0.05	> 0.05	> 0.05						
	Control	72.01 (1.19)	70.35 (0.88)	70.98 (1.59)	70.29 (1.49)	71.15 (1.13)	71.16 (0.86)									
OPP(mm Hg)	OHT	61.94 (1.62)	60.42 (1.51)	36.94 (1.34)	37.32 (1.38)	8.57 (1.71)	9.83 (2.26)	** < 0.001**	** < 0.001**	** < 0.001**	> 0.05	> 0.05	** < 0.001**	** < 0.001**	** < 0.001**	** < 0.001**
	Control	59.24 (1.00)	56.79 (1.25)	55.76 (1.49)	55.07 (1.16)	56.92 (0.98)	56.72 (0.79)									

Statistical analyses included main (within-groups and between groups) and interaction effects, with between-groups comparisons conducted at each condition both before and after FLS. A *p*-value < 0.05 was considered statistically significant

The bold indicates statistically signifi cant results

The OHT group exhibited significantly higher IOP and lower OPP in both LOP-1 and LOP-2 conditions (both pre- and post-FLS) compared to baseline values (both pre- and post-FLS), while no such changes were observed in the control group. Table 2 depicts Bonferroni-adjusted within-group comparisons from the mixed-model ANOVA for IOP and OPP, in the OHT and control groups along with their respective p-values.

**Table 2:** Results of Bonferroni-adjusted post-hoc comparisons (after mixed-model ANOVA) for IOP and OPP within the OHT and control groups at Pre-Loop (Baseline), LOP-1, and LOP-2 conditions, evaluated before and after FLS

Parameters	Group	Pre-Loop (Baseline) Before FLS versus vs Pre-Loop (Baseline) After FLS	Pre-Loop (Baseline) Before FLS versus vs Pre-Loop (Baseline) Before FLS	Pre-Loop (Baseline) Before FLS vs LOP-1 After FLS	Pre-Loop (Baseline) Before FLS vs LOP-2 Before FLS	Pre-Loop (Baseline) Before FLS vs LOP-2 After FLS	Pre-Loop (Baseline) After FLS vs LOP-1 Before FLS	Pre-Loop (Baseline) After FLS vs LOP-1 After FLS	Pre-Loop (Baseline) After FLS vs LOP-2 Before FLS	Pre-Loop (Baseline) After FLS vs LOP-2 After FLS	LOP-1 Before FLS vs LOP-1 After FLS	LOP-1 Before FLS vs LOP-2 Before FLS	LOP-1 Before FLS vs LOP-2 After FLS	LOP-1 After FLS vs LOP-2 Before FLS	LOP-1 After FLS vs LOP-2 After FLS	LOP-2 Before FLS vs LOP-2 After FLS
IOP(mm Hg)	OHT	> 0.05	** < 0.001**	** < 0.001**	** < 0.001**	** < 0.001**	** < 0.001**	** < 0.001**	** < 0.001**	** < 0.001**	> 0.05	** < 0.001**	** < 0.001**	** < 0.001**	** < 0.001**	** > 0.05**
	Control	> 0.05	> 0.05	> 0.05	> 0.05	> 0.05	> 0.05	> 0.05	> 0.05	> 0.05	> 0.05	> 0.05	> 0.05	> 0.05	> 0.05	> 0.05
OPP (mm Hg)	OHT	> 0.05	** < 0.001**	** < 0.001**	** < 0.001**	** < 0.001**	** < 0.001**	** < 0.001**	** < 0.001**	** < 0.001**	> 0.05	** < 0.001**	** < 0.001**	** < 0.001**	** < 0.001**	> 0.05
	Control	> 0.05	> 0.05	> 0.05	> 0.05	> 0.05	> 0.05	> 0.05	> 0.05	> 0.05	> 0.05	> 0.05	> 0.05	> 0.05	> 0.05	> 0.05

Statistical significance was set at p < 0.05

The bold indicates statistically signifi cant results

**Fig. 3:**
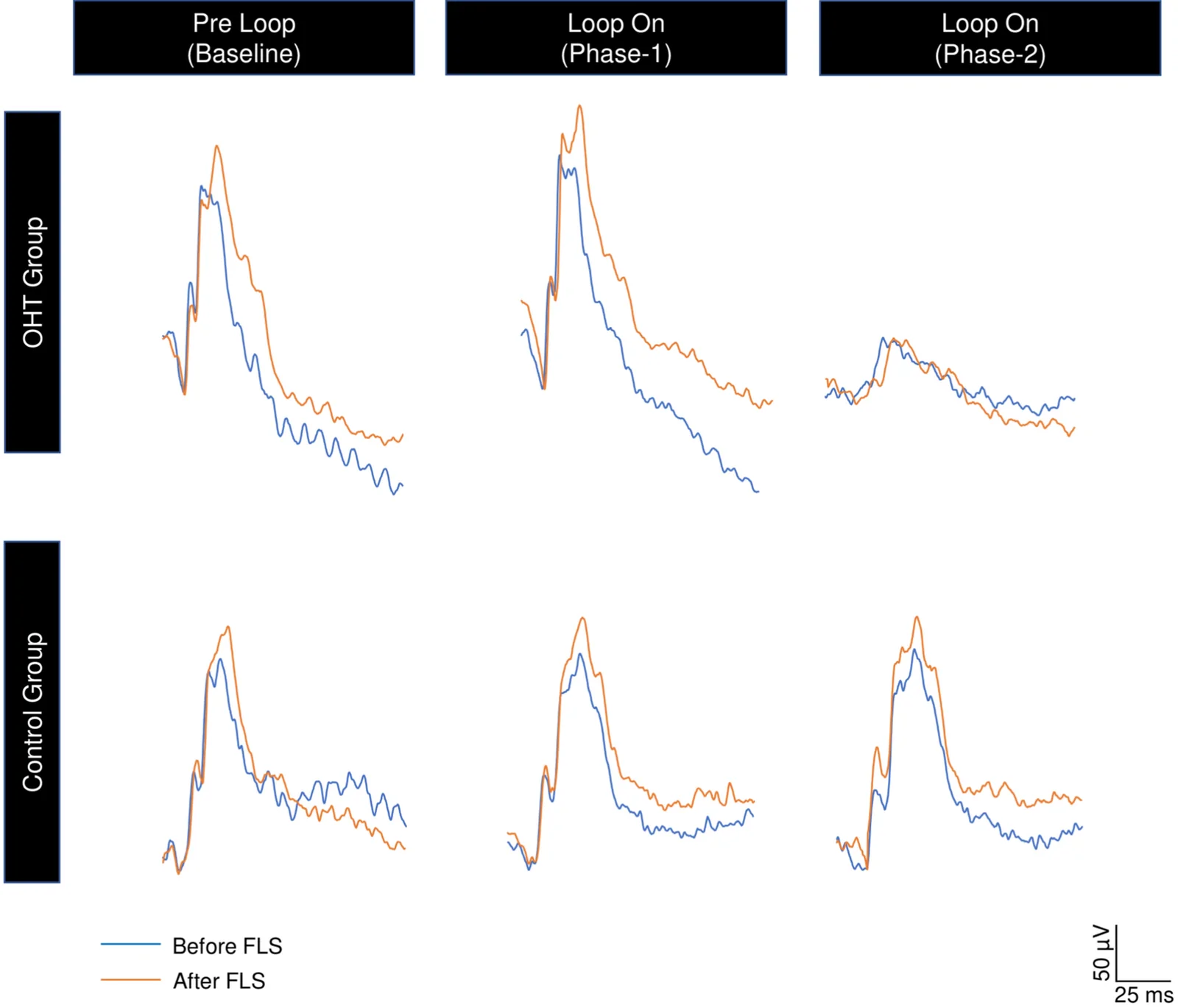
Representative traces of ffERG responses recorded before (blue line) and after (orange line) FLS from the OHT and control groups at three experimental conditions: Pre-Loop (Baseline), LOP-1, and LOP-2

Figure 3 depicts representative ffERG traces from one mouse of each experimental group, recorded at baseline (Pre-Loop), LOP-1, and LOP-2, with two traces (pre- and post-FLS) at each condition.

The mixed-model ANOVA showed significant within-group effects for all ffERG responses (amplitudes and implicit times of both the a-wave and b-wave). Significant between-group and interaction effects were detected for the amplitudes, but not for the implicit times, of the a-wave and b-wave. There were significant reductions in the amplitudes of both a-wave and b-wave at LOP-2 (both before and after FLS) in the OHT group when compared to the control group, with no corresponding changes in the implicit times. Table 3 shows the mean (SEM) of amplitudes and implicit times of the a-wave and b-wave of both experimental groups at baseline (Pre-Loop) and both Loop on phases conditions obtained before and after FLS, along with mixed-model ANOVA results and Bonferroni post-hoc comparisons between two groups. The supplementary material (ESM_2) contains the individual ffERG data recorded from both groups.

**Table 3:** Mean (SEM) values of the amplitudes (μV) and implicit times (ms) of the a-wave and b-wave measured before and after FLS in the OHT and control groups, at three conditions: Pre-Loop (Baseline), LOP-1, and LOP-2

ERG parameter	Group	Pre-loop (Baseline) before FLS	Pre-loop (Baseline) after FLS	LOP-1 before FLS	LOP-1 after FLS	LOP-2 before FLS	LOP-2 after FLS	Within-group main effect	Between groups main effect	Interaction Effect	Between groups comparisons (At)					
											Pre-Loop (Baseline) Before FLS	Pre-Loop (Baseline) After FLS	LOP-1 Before FLS	LOP-1 After FLS	LOP-2 Before FLS	LOP-2 After FLS
A-wave amplitude (µV)	OHT	15.44 (1.41)	15.73 (2.09)	17.75 (2.16)	20.15 (3.92)	8.10 (0.46)	7.88 (0.51)	** < 0.01**	** < 0.001**	** < 0.01**	> 0.05	> 0.05	> 0.05	> 0.05	** < 0.001**	** < 0.01**
	Control	17.66 (1.78)	18.40 (3.00)	19.22 (3.61)	19.00 (2.73)	17.52 (2.20)	19.05 (3.79)									
A-wave implicit time (ms)	OHT	18.74 (1.24)	20.12 (1.45)	24.61 (1.41)	23.91 (1.78)	29.61 (3.93)	28.98 (3.93)	** < 0.01**	> 0.05	> 0.05						
	Control	19.90 (0.79)	19.98 (1.27)	24.15 (1.03)	24.45 (1.59)	26.07 (1.28)	25.27 (1.38)									
B-wave amplitude (µV)	OHT	123.88 (3.79)	140.03 (5.41)	116.33 (11.12)	134.68 (11.99)	26.50 (2.90)	26.73 (2.83)	** < 0.001**	** < 0.01**	** < 0.001**	> 0.05	> 0.05	> 0.05	> 0.05	** < 0.001**	** < 0.001**
	Control	128.44 (11.29)	143.34 (12.23)	136.82 (14.03)	152.87 (14.02)	135.38 (15.87)	151.15 (17.67)									
B-wave implicit time (ms)	OHT	54.11 (2.86)	54.23 (3.08)	61.51 (2.77)	60.23 (2.96)	70.33 (2.23)	69.11 (2.14)	** < 0.001**	> 0.05	> 0.05						
	Control	64.11 (3.98)	62.81 (3.24)	67.01 (4.47)	67.55 (4.90)	69.25 (4.71)	72.06 (5.65)									

Statistical analyses included main effects (both within and between groups) and interaction effects, with between-group comparisons performed at each condition before and after FLS. Statistical significance was defined as a *p*-value < 0.05

The bold indicates statistically signifi cant results

The OHT group showed significant reductions in a-wave amplitudes (both pre- and post-FLS) at LOP-2 condition compared to both Pre-Loop (both before and after FLS) and LOP-1 (both before and after FLS) conditions. The implicit times of a-wave at both LOP-1(both before and after FLS) and LOP-2 (both before and after FLS) were significantly increased compared to Pre-Loop (before FLS), but no such delays were found in comparison to Pre-Loop (after FLS) condition. No significant changes in the amplitudes or implicit times of the a-wave were detected within the control group. The amplitudes of b-wave (after FLS) at Pre-Loop and LOP-1 conditions were significantly increased compared to corresponding b-wave amplitudes (before FLS) in both OHT and control groups. Following FLS, there was a significant increase in b-wave amplitudes at LOP-2 in the control group, but not in the OHT group. The implicit times of b-wave were significantly increased at LOP-2 (both pre- and post-FLS) compared to Pre-Loop (both pre- and post-FLS) in the OHT group. There were no significant changes in the implicit times within the control group. Table 4 presents Bonferroni-adjusted within-group comparisons of ffERG responses from the mixed-model ANOVA for both experimental groups.

**Table 4:** Results of Bonferroni-adjusted post-hoc comparisons (after mixed-model ANOVA) for the amplitudes and implicit times of the a-wave and b-wave within the OHT and control groups at Pre-Loop (Baseline), LOP-1, and LOP-2 conditions, measured before and after FLS

ERG responses	Group	Pre-loop (Baseline) before FLS versus Pre-loop (Baseline) after FLS	Pre-loop (Baseline) before FLS versus LOP-1 before FLS	Pre-loop (Baseline) before FLS versus LOP-1 after FLS	Pre-loop (Baseline) before FLS versus LOP-2 before FLS	Pre-loop (Baseline) before FLS versus LOP-2 after FLS	Pre-Loop (Baseline) After FLS vs LOP-1 Before FLS	Pre-Loop (Baseline) After FLS vs LOP-1 After FLS	Pre-Loop (Baseline) After FLS vs LOP-2 Before FLS	Pre-Loop (Baseline) After FLS vs LOP-2 After FLS	LOP-1 Before FLS vs LOP-1 After FLS	LOP-1 Before FLS vs LOP-2 Before FLS	LOP-1 Before FLS vs LOP-2 After FLS	LOP-1 After FLS vs LOP-2 Before FLS	LOP-1 After FLS vs LOP-2 After FLS	LOP-2 Before FLS vs LOP-2 After FLS
A-wave amplitude (µV)	OHT	> 0.05	> 0.05	> 0.05	** < 0.05**	** < 0.05**	> 0.05	> 0.05	** < 0.05**	** < 0.05**	> 0.05	> 0.05	** < 0.01**	> 0.05	** < 0.05**	> 0.05
	Control	> 0.05	> 0.05	> 0.05	> 0.05	> 0.05	> 0.05	> 0.05	> 0.05	> 0.05	> 0.05	> 0.05	> 0.05	> 0.05	> 0.05	> 0.05
A-wave implicit time (ms)	OHT	> 0.05	** < 0.01**	** < 0.05**	** < 0.05**	** < 0.05**	> 0.05	> 0.05	> 0.05	> 0.05	> 0.05	> 0.05	> 0.05	> 0.05	> 0.05	> 0.05
	Control	> 0.05	> 0.05	> 0.05	> 0.05	> 0.05	> 0.05	> 0.05	> 0.05	> 0.05	> 0.05	> 0.05	> 0.05	> 0.05	> 0.05	> 0.05
B-wave amplitude (µV)	OHT	** < 0.001**	> 0.05	> 0.05	** < 0.001**	** < 0.001**	> 0.05	> 0.05	** < 0.001**	** < 0.001**	** < 0.001**	** < 0.001**	** < 0.001**	** < 0.001**	** < 0.001**	> 0.05
	Control	** < 0.01**	> 0.05	> 0.05	> 0.05	> 0.05	> 0.05	> 0.05	> 0.05	> 0.05	** < 0.01**	> 0.05	> 0.05	> 0.05	> 0.05	** < 0.001**
B-wave implicit time (ms)	OHT	> 0.05	> 0.05	> 0.05	** < 0.01**	** < 0.05**	> 0.05	> 0.05	** < 0.01**	** < 0.05**	> 0.05	> 0.05	> 0.05	> 0.05	> 0.05	> 0.05
	Control	> 0.05	> 0.05	> 0.05	> 0.05	> 0.05	> 0.05	> 0.05	> 0.05	> 0.05	> 0.05	> 0.05	> 0.05	> 0.05	> 0.05	> 0.05

Statistical significance was determined at *p* < 0.05

The bold indicates statistically signifi cant results

**Fig. 4:**
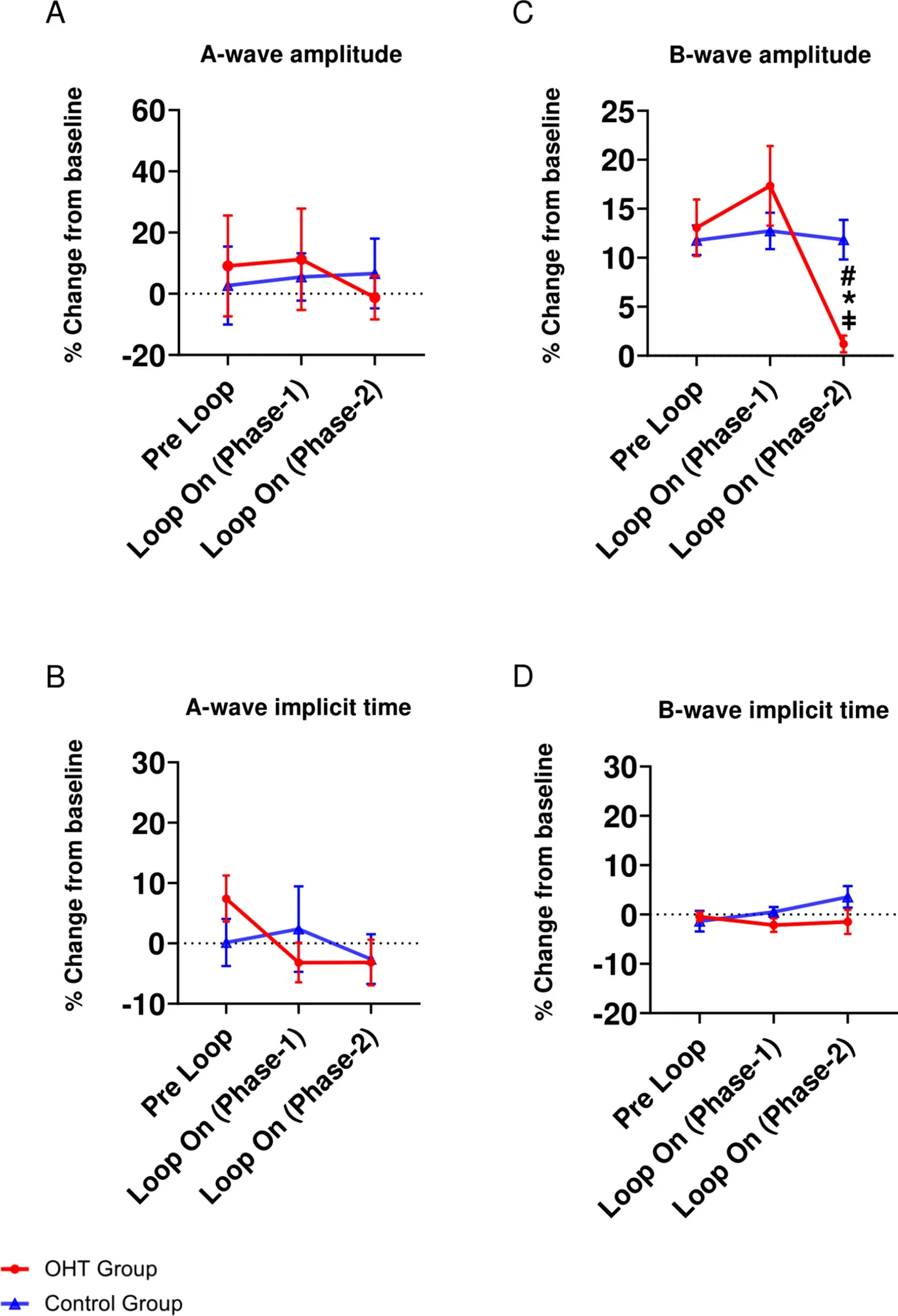
Mean percentage changes (from corresponding baseline at Pre-Loop, LOP-1, and LOP-2 in a-wave amplitude **A**, a-wave implicit time **B**, b-wave amplitude **C**, and b-wave implicit time **D**, respectively. Error bars represent standard error of the mean (SEM). The red line with circle represents the “OHT Group”, while the blue line with triangle represents the “Control Group”. % change from baseline = [ {(After FLS–Before FLS)/ Before FLS} × 100]. ^‡^*p* < 0.001: Pre-Loop versus LOP-2 in the OHT group, *p < 0.01: LOP-1 versus LOP-2 in the OHT group, and.^#^*p* < 0.001: [ Control group vs. OHT group at LOP-2]

Figure 4 shows that there were significant differences in percentage changes in b-wave amplitudes (within-group: *p* < 0.001; between groups: *p* < 0.05; interaction effect: *p* = 0.007). The percentage changes in b-wave amplitudes did not differ significantly between the two groups at the Pre-Loop and LOP-1 conditions (*p* > 0.05). However, at the LOP-2 condition, the OHT group exhibited a significantly smaller percentage change in b-wave amplitudes compared to the control group (*p* < 0.001) as shown in Fig. 4. The percentage change in b-wave amplitudes at LOP-2 was significantly reduced compared to the Pre-Loop and LOP-1 conditions (both *p* < 0.001). However, there were no significant differences in the percentage increases in b-wave amplitudes within the control group (Pre-Loop vs. LOP-1: *p* > 0.05; Pre-Loop vs. LOP-2: *p* > 0.05; LOP-1 vs. LOP-2: *p* > 0.05). The percentage changes in a-wave amplitudes (mixed model ANOVA: within-group, *p* > 0.05; between groups, *p* > 0.05; interaction effect, *p* > 0.05), a-wave implicit times (mixed model ANOVA: within-group, *p* > 0.05; between groups, *p* > 0.05; interaction effect, *p* > 0.05), and b-wave implicit times (mixed model ANOVA: within-group, *p* > 0.05; between groups, *p* > 0.05; interaction effect, *p* > 0.05) were not significantly different between the two groups or within groups.

## Discussion

To our knowledge, this is the first study to assess the short-term impacts of moderate (~ 35 mm Hg) and high (~ 65 mm Hg) IOP elevations on flicker-evoked changes in ffERG waveforms in a murine model. We previously reported that FLS for 60 s at 12 Hz (0.1 cd·s/m^2^) significantly enhanced both arterial and venous blood flow, as well as the b-wave amplitude (compared to the corresponding baseline) in C57BL/6 J mice [17]. However, mice were subjected to FLS for 160 s in the present study. This duration was selected based on our recent findings (unpublished data) that 160 s of FLS induces significantly higher increase in arterial and venous blood flow compared to 60 s.

Anatomically, the MRL comprises Müller cells, horizontal cells, bipolar cells, and amacrine cells [23]. In C57BL/6 J mice, Müller cells are the principal retinal glial cells, providing structural, metabolic, and neuroprotective support to maintain retinal homeostasis [24]. Horizontal cells, coupled via gap junctions, receive glutamatergic input from photoreceptors and provide feedback to photoreceptors and feedforward to bipolar cells [25, 26]. Amacrine cells modulate, integrate and refine visual signals between bipolar and ganglion cells [27]]. They contribute to directional motion, scotopic vision, noise reduction, light/dark adaptation and saccade blurring suppression [27]. Bipolar cells are interneurons that process and relay signals from photoreceptors to ganglion cells and amacrine cells [28, 29]. The ERG b-wave originates in the MRL and is largely driven by the activity of bipolar cells [28, 29]. Given the low background luminance used in our study, the recorded ERG b-wave reflects the integrated responses of both rod and cone bipolar cells [28, 29]. Hence, the enhanced b-wave amplitude obtained after FLS (compared to the corresponding baseline) is likely primarily driven by the increased electrical activity of both rod and cone bipolar cells.

The current study demonstrated that transient FLS significantly enhanced the ffERG b-wave amplitudes compared to the corresponding baseline, reflecting increased activity in the MRL layer, even under moderate IOP elevation of ~ 35 mm Hg. However, such enhancement was not observed at the high IOP elevation of approximately 65 mm Hg. Although an IOP value of ~ 35 mm Hg seems moderate compared to other levels in our study, it is important to note that this value is approximately three times higher than the corresponding baseline of around 12 mm Hg. When compared to their corresponding baselines, the b-wave amplitudes increased by more than 12% and 15% at the Pre-Loop and LOP-1 conditions, respectively, but the percentage change in b-wave amplitude was significantly reduced in the OHT group at the LOP-2 condition. In contrast, the control group showed consistent increases of 11–13% across all conditions. These findings suggest that the retina can effectively adapt to short-term moderate IOP elevation, but not to high levels, sustaining a normal increase in ffERG b-wave amplitudes in response to the FLS.

The significant increases of b-wave amplitudes following FLS at baseline and LOP-1 in the OHT group, as well as at all time points in the control group, align with the findings of our recent study [17]. Our findings revealed that short-term moderate IOP elevation of 35 mm Hg did not impair the retinal physiology to enhance electrical activity, as shown by increased b-wave amplitudes (compared to the corresponding baseline). This implies some degree of retinal resilience or adaptive capacity to a transient and moderate IOP elevation. In contrast, short-term high IOP elevation of 65 mm Hg almost completely abolished the flicker-induced enhancement of the b-wave. This indicates that the retina cannot sustain increased ERG responses (compared to the corresponding baseline) after FLS as the IOP above certain threshold would cause retinal physiological impairment. Previous studies have reported the upregulation of heme oxygenase-1 and glial fibrillary acidic protein in retinal microglia [30], along with significant thinning of the middle and inner retinal layers after acute IOP elevation [31], indicating retinal physiological and structural impairment. However, the threshold IOP and the duration of IOP elevation required to induce these changes are still not fully understood. Tan et al. reported that IOP elevation to 45 mm Hg for 30 min significantly increased single flash ERG a-wave and b-wave amplitudes [32]. These increased amplitudes persisted even after IOP normalization following loop removal. However, significant reductions in retinal functional responses (second harmonic component of ERG response) and total RBF in response to FLS were observed when compared to normal IOP conditions in rats. In contrast, they also mentioned that further elevation of IOP beyond 60 mm Hg resulted in progressive reduction of a-wave and b-wave amplitudes. In line with their results, significant reductions were noted in both a-wave and b-wave amplitudes at around 65 mm Hg in the current study. Notably, apart from the differences in IOP levels and durations of IOP elevation, the flickering frequency and stimulation duration were not consistent between their study (10 Hz for 2 s) and our study (12 Hz for 160 s). Choh et al. recently reported enhanced ffERG a-wave and b-wave amplitudes when IOP was elevated to the moderate level of 35 mm Hg, using similar techniques utilized in our study [33]. However, our findings showed no significant changes in the baseline (Pre-Loop) a-wave and b-wave amplitudes compared to LOP-1 at similar IOP levels (~ 35 mm Hg). A key difference between the two studies may be the photopic ffERG measured in mice in our study, whereas Choh et al. recorded scotopic ffERG in rats. These differences could influence the changes in flicker-induced ERG responses. In addition, our study showed a significant increase in b-wave amplitudes after FLS at ~ 35 mm Hg, while b-wave amplitudes measured after FLS were not significantly different from the corresponding baseline (before FLS) at ~ 65 mm Hg. Numerous studies have demonstrated that prolonged chronic IOP elevation below 35 mm Hg can lead to increased nitric oxide production in the retina and optic nerve [34–36]. Further investigation is warranted to explore the effects of flickering light on nitric oxide production under varying acute IOP conditions. In our study, while BP remained consistent in both groups, there was a significant reduction in OPP in the OHT group. Specifically, OPP decreased from 60 to 62 mm Hg at the Pre-Loop condition to 36–37 mm Hg at LOP-1, and further diminished to 8–10 mm Hg at LOP-2 condition. Previous studies have shown that a severe reduction in OPP could disrupt vascular autoregulation and alter neurovascular coupling in the retina [32, 37]. Tan et al. reported reduced flicker-evoked retinal blood flow (RBF) when the OPP was ~ 55 mm Hg in rats [32]. Similarly, Riva et al. also reported no significant increase in RBF when OPP was ~ 20 mm Hg in cats, which is about twice the OPP induced in LOP-2 condition of our study [37]. These studies suggest that retinal neurovascular coupling is preserved until OPP decreases beyond a certain threshold. Although the current study did not measure RBF in response to FLS, it can be speculated that reduced OPP may lead to insufficient nutrients and oxygen supply to the retina [38]. Consequently, this could severely impact the metabolism of retinal neurons, making them less responsive to FLS, as indicated by the loss of enhancement in ffERG responses following FLS. This speculation is supported by our previous work, where we observed a significant positive correlation between increased RBF and enhanced b-wave amplitudes following FLS in the same strain of mice [17]. Furthermore, elevated IOP induces direct mechanical damage, disrupting cellular homeostasis and diminishing retinal functional responses. There were several limitations of this study. First, the tightening of the loop might have exerted excessive compressive forces from the exterior inward during model induction. This may differ from real ocular hypertensive conditions, where IOP increases due to elevated pressure originating from within the eyeball, exerting an outward force. Second, we did not measure any corresponding changes in retinal hemodynamic components, such as RBF and vessel diameters. Third, while the target IOP was set at 35 mm Hg and 65 mm Hg in each mouse under LOP-1 and LOP-2 conditions respectively in the OHT group, the observed IOP levels varied due to manual adjustments of the loop, resulting in fluctuations that prevented precise IOP targets.

Based on the findings of the present study, future studies can be conducted. Since the present study was limited to just two IOP levels, subsequent studies should investigate the effects of a broader range of IOP levels on flicker-induced changes in retinal electrical activity. Moreover, future investigations should assess how hemodynamic components are altered in response to FLS under these varying IOP levels. Additional research should explore whether the changes observed under acutely elevated IOP conditions are also relevant in acute attack glaucoma conditions. Future studies can be conducted to explore the effects of OPP modulation on flicker-induced changes in retinal electrical activity and hemodynamic components such as RBF, blood flow velocity, and vessel diameter. This would further enhance our understanding of the relationship between IOP, BP, OPP, and vascular autoregulation within the context of retinal neurovascular coupling.

In conclusion, flicker-induced transient enhancement in b-wave amplitude remained consistent during short-term IOP elevation to approximately 35 mm Hg, indicating robust retinal adaptability to moderate IOP elevation, despite a significant reduction in OPP. However, the short-term IOP elevation to approximately 65 mm Hg caused a significant reduction in the flicker-induced enhancement of b-wave amplitude. This suggests that mechanical stress caused by high IOP with a severe reduction in OPP surpasses the ability of the retina to adapt to FLS and causes a physiological impairment.

## Commercial relationship disclosures

Milan Rai: None, Xiayin Yang: None, Kai Yip Choi: None, Shaoying Tan: None, Henry Ho-lung Chan: None.

## Supplementary Information

Below is the link to the electronic supplementary material.

Supplementary file1 (XLSX 13 KB)

## Data Availability

Data is provided within the manuscript or supplementary information files.

## Declarations

### Statement on the welfare of animals

All applicable international, national and/or institutional guidelines for the care and use of animals were followed. All procedures performed in studies involving animals were in accordance with the ethical standards of the institution or practice at which the studies were conducted.
